# Prevalence of SARS-CoV-2 infection and associated risk factors: A testing program and nested case-control study conducted at Sapienza University of Rome between March and June 2021

**DOI:** 10.3389/fpubh.2022.1010130

**Published:** 2022-10-19

**Authors:** Valentina Baccolini, Leonardo Maria Siena, Erika Renzi, Giuseppe Migliara, Corrado Colaprico, Alessandra Romano, Azzurra Massimi, Carolina Marzuillo, Corrado De Vito, Leandro Casini, Guido Antonelli, Ombretta Turriziani, Antonio Angeloni, Fabrizio D'Alba, Paolo Villari, Antonella Polimeni, Collaborating Group, Federica Maria Di Lella

**Affiliations:** ^1^Department of Public Health and Infectious Diseases, Sapienza University of Rome, Rome, Italy; ^2^Special Office for Prevention, Protection and High Vigilance, Sapienza University of Rome, Rome, Italy; ^3^Department of Molecular Medicine, Sapienza University of Rome, Rome, Italy; ^4^Department of Experimental Medicine, Sapienza University of Rome, Rome, Italy; ^5^Policlinico Umberto I Teaching Hospital, Rome, Italy; ^6^Department of Oral and Maxillofacial Science, Sapienza University of Rome, Rome, Italy

**Keywords:** COVID-19, testing program, university, case-control study, risk factors, students

## Abstract

**Background:**

To safely resume in-person activities during the COVID-19 pandemic, Sapienza University of Rome implemented rigorous infection prevention and control measures, a successful communication campaign and a free SARS-CoV-2 testing program. In this study, we describe the University's experience in carrying out such a program in the context of the COVID-19 response and identify risk factors for infection.

**Methods:**

Having identified resources, space, supplies and staff, from March to June 2021 Sapienza offered to all its enrollees a molecular test service (8.30 AM to 4 PM, Monday to Thursday). A test-negative case-control study was conducted within the program. Participants underwent structured interviews that investigated activity-related exposures in the 2 weeks before testing. Multivariable conditional logistic regression analyses were performed. Adjusted odds ratios (aORs) and 95% confidence intervals (95% CIs) were calculated.

**Results:**

A total of 8,959 tests were administered, of which 56 were positive. The detection trend followed regional tendencies. Among 40 cases and 80 controls, multivariable analysis showed that a known exposure to a COVID-19 case increased the likelihood of infection (aOR: 8.39, 95% CI: 2.38–29.54), while having a job decreased it (aOR: 0.23, 95% CI: 0.06–0.88). Of factors that almost reached statistical significance, participation in activities in the university tended to reduce the risk (aOR: 0.32, 95% CI: 0.09–1.06), while attendance at private gatherings showed an increasing risk trend (aOR: 3.48, 95% CI: 0.95–12.79). Age, gender, activities in the community, visiting bars or restaurants, and use of public transportation were not relevant risk factors. When those students regularly attending the university campus were excluded from the analysis, the results were comparable, except that attending activities in the community came close to having a statistically significant effect (aOR: 8.13, 95% CI: 0.91–72.84).

**Conclusions:**

The testing program helped create a safe university environment. Furthermore, promoting preventive behavior and implementing rigorous measures in public places, as was the case in the university setting, contributed to limit the virus transmission.

## Introduction

The COVID-19 pandemic has created considerable disruption to education systems all over the world (1). The temporary physical closures of schools that occurred, and continue to occur, in many countries, including Italy, have left policymakers and educational institutions with unprecedented challenges (2). Most universities switched to emergency remote teaching to attempt to mitigate learning losses (3). Despite initial problems relating to technology, tools and training for both staff and students (2), progress was made during the digital transition (4), but how to safely reopen educational institutions quickly became a major concern (5, 6). To be able to resume in-person activities, many universities developed a COVID-19 Task Force with an illness response procedure and applied a health protocol that included social distancing and face-mask use; some even offered an optional on-campus testing program to identify and isolate cases promptly (7, 8).

While case detection and contact tracing are critical for effectively containing the pandemic and understanding epidemiological trends (9), the identification of risk factors for SARS-CoV-2 acquisition helps to better guide ongoing mitigation efforts and inform policy-makers (10). In line with ecological studies that have identified population density, overcrowding and mobility as relevant infection determinants (11), many countries have introduced strict and wide-reaching measures (12), which reduced virus transmission to low levels by the spring and summer of 2020 (13). Nevertheless, during the following fall, the same countries witnessed a continued resurgence in cases that forced them to apply various degrees of curfew (14). Given the social and economic impact of these stay-at-home orders (15), determining the settings with the highest risk of SARS-CoV-2 transmission has become a priority, particularly because a growing proportion of cases has no clear epidemiological link (16).

In the Italian context, Sapienza University of Rome has been deeply committed to creating a safe learning and working environment during the pandemic. Following the introduction of national and international guidelines, the University was very quick to implement measures for risk prevention and management of COVID-19 and, starting from March 2021, has offered free SARS-CoV-2 tests to all its enrollees. Given that students have high levels of social interaction and mobility and exhibit a disease profile that is often asymptomatic or shows few symptoms (17), such a testing program became a valuable opportunity to investigate potential exposure associated with virus transmission. In this study, we aimed to (i) describe the University's experience of implementing a voluntary SARS-CoV-2 testing program from March to June 2021 in the context of the COVID-19 response, and (ii) to identify risk factors for virus acquisition in the student population through a nested case-control study.

## Materials and methods

### Testing program

Sapienza University of Rome is among the largest European universities, with more than 100,000 students enrolled in the 2020-21 academic year (18). Early in 2021, the Sapienza University governance started a discussion of possible ways of providing COVID-19 testing to students who were required to be on campus for in-person activities. Financial resources were mobilized, suitable locations were identified, and the necessary supplies, information technology systems and staff were put in place. The University teams involved included the Special Office for Prevention, Protection and High Vigilance, the Department of Public Health and Infectious Diseases, the Department of Molecular Medicine, and the Department of Experimental Medicine.

Thanks to a collaboration with the Lazio Region and Policlinico Umberto I General Hospital, from 1 March to 30 June 2021, the University employed a voluntary COVID-19 testing program. All students enrolled in 2020-21 Sapienza degree programs were offered a RT-PCR (reverse transcriptase-polymerase chain reaction) molecular test free of charge in front of the Ciao-Hello offices at the main campus. The invitation was sent *via* institutional e-mail. The collection method used was a nasal swab test. This service ran on Monday-Friday from 8.30 AM to 4 PM for the first 2 weeks. It was then paused when the University was closed because of COVID-19 regional restrictions and Easter holidays, restarting again on 12 April 2021, and running Monday-Thursday with the same opening hours until the end of June. It was possible for a student to take more than one test throughout the campaign, provided that seven days had passed from the previous test. In addition, students were allowed to book a SARS-CoV-2 test only if asymptomatic and not under quarantine or isolation restrictions. The testing appointment schedule allowed for 20 appointments every 30 min (40 appointments/h), with a maximum capacity of 300 tests/day.

Every morning, the testing center staff arrived 30 min before the start of testing to collect personal protective equipment and participate in a safety briefing. The staff consisted of three people from the administration, in charge of the registration procedures and informed consent signatures, and one registered nurse, who acted as the supervisor responsible for sample collection. In addition, for each daily shift (one in the morning and one in the afternoon), four undergraduate nursing students worked 2-week rotations at the testing center as part of their semester clinical course. Three other students who were enrolled in the “Prevention Techniques” degree course spent part of their internship providing information and support to students waiting to be tested. All students involved in the testing center activities received specific orientation, education and training about the program during an online seminar on 25 February 2021.

A courier picked up the specimens collected at the testing center three times a day, at 11 AM, 2 PM and 4 PM, and transported them to the laboratories of Azienda Policlinico Umberto I General Hospital. Test results were available for student download from the Regione Lazio website 24–36 h after testing. Positive students were reported to the Department of Public Health and Infectious Diseases, which was delegated by the Rome Local Health Unit 1 to conduct contact tracing. Contact tracers interviewed infected students to identify close contacts during the 48 h prior to the test and collected exposure details, including dates, proximity, location, duration of exposure, and mask use.

### Case-control study

A test-negative case-control study (19) was conducted within the testing program to identify risk factors for SARS-CoV-2 acquisition. Each case detected was matched to two controls randomly selected from students who tested negative on the same day as the positive case. The control selection process was carried out by generating a random number sequence using the free RNG software, available at https://it.piliapp.com/random/number/. Specifically, for each case, five potential controls were initially selected; these were then contacted in the order of identification until two students had agreed to be recruited. Then, ~2–3 days after their test result, both cases and controls underwent structured 10-min interviews in Italian or English. They were asked 22 questions grouped in three different sections.

The first section collected sociodemographic information: age, gender, nationality, Italian region (if applicable), faculty, year of study, and job (if applicable). We also explored whether they had a chronic condition or were living with someone with a chronic condition.

In the second section, we asked participants to rate from 1 (never) to 5 (always) how frequently they had worn a mask indoors and had performed hand-hygiene procedures in the 2 weeks before testing. We also investigated whether they had had a known exposure to someone with COVID-19 or with signs/symptoms suggestive of COVID-19, defined as being within two meters for a total of ≥15 min without any mask within 24 h.

The last section explored potential exposures that had occurred in the 2 weeks prior to the swab. Participants were asked to express on a 5-point Likert-type scale from “never” to “more than once per day” or “always” how often they, on average, attended activities inside the university campus (e.g., lectures, internships); visited bars or restaurants within the university campus or outside the university campus (e.g., breakfast, lunch, aperitif, dinner or after dinner); visited cinemas, theaters, museums or churches; visited a salon or aesthetic centers, shopping centers, or grocery stores; participated in volunteer activities or courses outside the university (e.g., painting or photography courses); had guests or were guests, or attended private social or religious gatherings (e.g., parties, ceremonies); participated in indoor sport activities (e.g., gym, swimming pool); and visited healthcare facilities (general practitioner, hospital). We also asked them whether they had used public transportation for either short (within the city) or long distances.

The study was performed in accordance with the World Medical Association Declaration of Helsinki. Participants were asked for their consent and were guaranteed anonymity in the information collected. The institutional ethics board of the Umberto I teaching hospital/Sapienza University of Rome approved this study (protocol 188/2021).

### Statistical analysis

Data on regional and national detection rates of confirmed SARS-CoV-2 infections were collected from the Italian Civil Protection/Ministry of Health website (20).

Descriptive statistics were obtained using median and interquartile range or mean and standard deviation for continuous variables, and proportions for dichotomous and categorical variables. For the purposes of this analysis, students were considered as Italian vs. non-Italian; the Italian regions were grouped into two groups (Lazio vs. others); faculties were categorized into healthcare (e.g., medicine, nursing), science (e.g., mathematics, biology) or other (e.g., law, economics); self-reported adherence to hand-washing procedures and mask wearing were collapsed into two modalities (always/often vs. sometimes/rarely/never); and exposure activity responses during the 14 days before testing for SARS-CoV-2 were dichotomized as never vs. once or more.

Each variable was first examined through univariable conditional logistic regression analysis. Then, a multivariable conditional logistic regression model was built to identify predictors of SARS-CoV-2 infection. Variables were included in the model based on expert opinion. Given the limited sample size, we only adjusted the potential exposures for sex, age, having a job, and known exposure to a COVID-19 case or someone with sign/symptoms suggestive of COVID-19. Additionally, we collapsed the potential exposures into five categories: activities in the community (including essential and non-essential activities), activities in the university, going to bar or restaurants (within or outside the campus), use of public transportation (for short or long distances), and having or being guests/attending social or religious gatherings (dichotomous). As a result, the final model consisted of the following variables: age (continuous), gender (dichotomous), having a job (dichotomous), known exposure to a COVID-19 case or someone with signs/symptoms suggestive of COVID-19 (dichotomous), activities in the community (dichotomous), activities in the university (dichotomous), bars or restaurants (dichotomous), use of public transportation (dichotomous), and having or being guests/attending private social or religious gatherings (dichotomous). Adjusted odds ratios (aORs) and 95% confidence intervals (CIs) were calculated. A second conditional logistic regression model was restricted to participants who did not report attending the university campus in the 2 weeks before testing (29 cases matched to 42 controls in a 1:1 or 1:2 ratio). The same variables used in the first analysis were considered.

All calculations were performed using Stata (StataCorp LLC, 4905 Lakeway Drive, College Station, TX 322, USA), version 17.0. A two-sided *p* < 0.05 was considered statistically significant.

## Results

### Testing program

**Figure 1:**
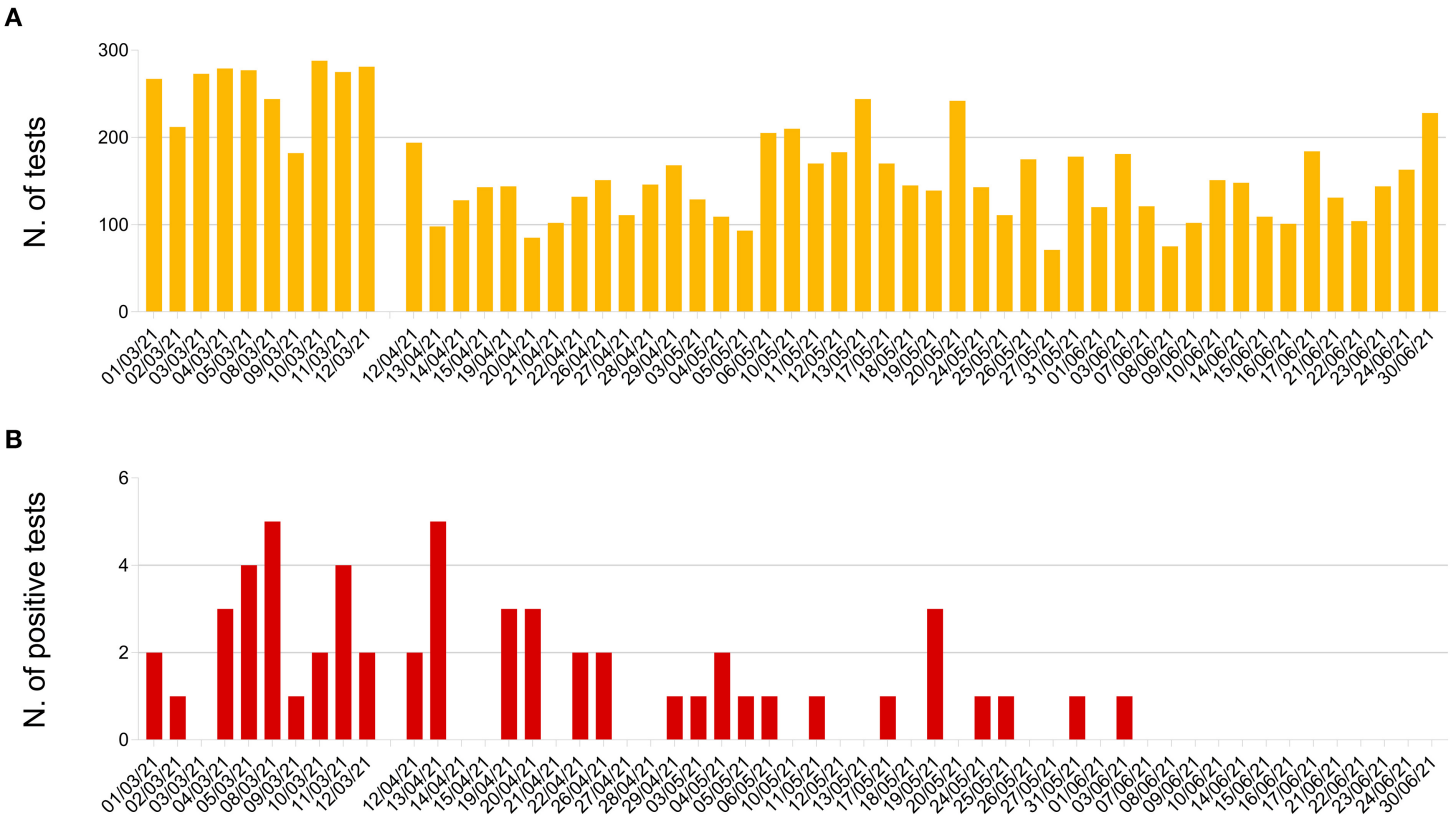
Sapienza University testing program, 1 March-30 June 2021: **(A)** Number of SARS-CoV-2 RT-PCR tests administered; **(B)** number of positive tests detected.

During the 14-week testing program, 9,982 reservations were made and 8,959 SARS-CoV-2 RT-PCR tests were administered (89.8%), with the first 2 weeks registering the highest number of participants (daily mean: 257.8; range: 182–288) (Figure 1A). A total of 56 students (0.63%) tested positive throughout the program; 24 (42.9%) of these were in the period 1 to 12 March 2021 (daily mean: 2.4; range: 0–5) (Figure 1B).

**Figure 2:**
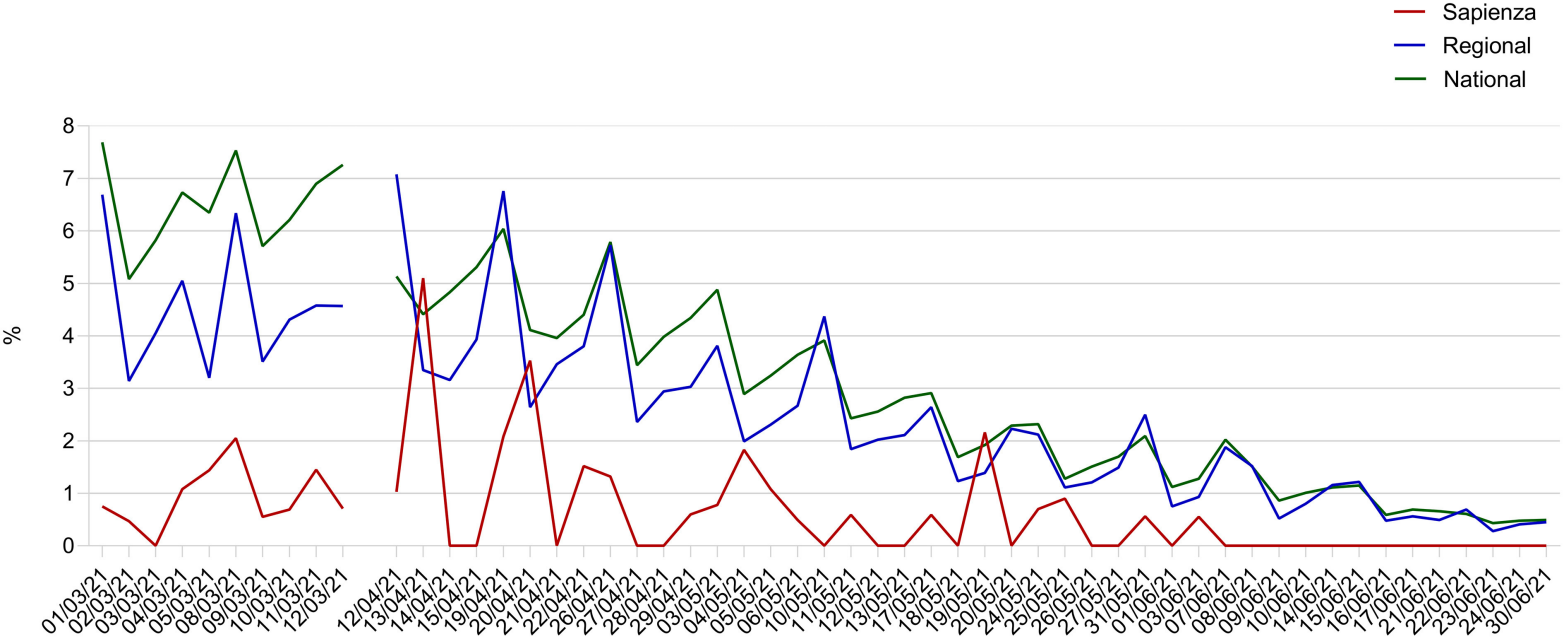
Daily detection rates of SARS-CoV-2 RT-PCR positive tests registered through the Sapienza University testing program (1 March-30 June 2021) in comparison to regional and national rates of COVID-19 confirmed cases.

Overall, the trend of our daily detection rate was comparable to those registered both at national and regional level, with the highest proportion of cases registered in the first 4 weeks of activity and a clear reduction in the following days (Figure 2). However, apart from a few fluctuations, our proportions were always lower, ranging mostly from 0 to 2% and exceeding this level on only a few occasions (8 March, 13 April, 20 April, and 20 May).

As for the sociodemographic characteristics of the students tested, out of 6,924 students who participated at least once in the program, the vast majority were females (61.3%) and Italian (94.3%) (Table 1). Mean age was 23.9 years (± 4.9 years). The largest category was students enrolled in faculties not related to healthcare or science (~40%). Lastly, approximately three quarters of the students had a high academic level (third year or above).

**Table 1:** Characteristics of students who were tested at least once for SARS-CoV-2 at Sapienza University of Rome from 1 March to 30 June 2021 (*N* = 6,924).

	***N* (%)**
**Gender**	
Female	4,245 (61.3)
Male	2,679 (38.7)
**Age, years (*****N*** **=** **6,922)**	
Mean (SD)	23.9 (4.9)
Median (IQR)	23 (21–25)
**Country of residence**	
Italy	6,526 (94.3)
Others	278 (4.0)
Missing	120 (1.7)
**Area of study**	
Healthcare	2,139 (30.9)
Science	1,855 (26.8)
Other	2,788 (40.2)
Missing	142 (2.1)
**Year of study**	
First	751 (10.9)
Second	1,228 (17.7)
Third	1,505 (21.7)
Fourth	1,081 (15.6)
Fifth	1,471 (21.2)
Sixth	208 (3.0)
Master degree, doctorate degree, specialization school	408 (5.9)
Outside prescribed course	130 (1.9)
Missing	142 (2.1)

Results are expressed as mean (standard deviation, SD), median (interquartile range, IQR), or frequency (percentage).

### Case-control study

Out of 56 positives cases detected during the testing program, 11 students refused to participate in the study, whereas another five did not speak fluent Italian or English, leaving a total of 40 individuals who were interviewed (response rate: 71.4%). Similarly, six potential controls declined to be enrolled, accounting for a control response rate of ~93%.

Cases and controls were mostly female (65.0 vs. 72.5%) and had a similar age on average (22.2 vs. 23.3 years) (Table 2). The majority in both groups came from the Lazio region (60.0 vs. 61.3%). Regarding their academic curricula, study participants mostly attended the first 2 years of university (57.5 vs. 63.8%), mainly in non-health-related faculties (82.5% both), but more students in the control group were employed at the time of the interview (12.5 vs. 28.8%). Approximately one third of the students had a chronic condition or were living with someone with a chronic condition (35.0 vs. 33.8%). Whereas, all students in both groups self-reported as having always or often worn a mask in the 2 weeks before testing, not all cases had frequently performed hand-hygiene procedures compared to controls (90 vs. 100%). Almost half of the cases reported having had a contact with a COVID-19 case or someone with signs and/or symptoms suggestive of COVID-19 compared to 12.5% of controls.

**Table 2:** Students' sociodemographic characteristics and self-reported adherence to precautionary measures.

	**Cases**	**Controls**	Unadjusted OR (95% CI) *	*p*-Value*
Gender				
Male	14 (35.0)	22 (27.5)	Ref.	
Female	26 (65.0)	58 (72.5)	0.74 (0.35–1.58)	0.432
Age, years			0.90 (0.80–1.02)	0.111
Mean (SD)	22.2 (2.8)	23.3 (3.8)		
Median (IQR)	22 (20–24)	22 (21–25)		
Region of residence				
Lazio	24 (60.0)	49 (61.3)	Ref.	
Others	16 (40.0)	31 (38.8)	0.95 (0.43–2.08)	0.894
Area of study				
Healthcare	7 (17.5)	14 (17.5)	Ref.	
Others	33 (82.5)	66 (82.5)	1.00 (0.36–2.74)	0.999
Year of study				
First	11 (27.5)	22 (27.5)	Ref.	
Second	12 (30.0)	29 (36.3)	0.83 (0.30–2.28)	0.719
Third	10 (25.0)	11 (13.8)	1.69 (0.56–5.12)	0.351
Fourth or above	7 (17.5)	18 (22.5)	0.77 (0.24–2.51)	0.666
Having a job	5 (12.5)	23 (28.8)	0.38 (0.13–1.05)	0.063
Having a chronic condition or living with someone with a chronic condition	14 (35.0)	27 (33.8)	1.06 (0.50–2.39)	0.890
Mask use indoors ≤ 14 days before SARS-CoV-2 test			–	
Always, often	40 (100.0)	80 (100.0)		
Sometimes, rarely, never	0 (0.0)	0 (0.0)		
Hand-hygiene procedures ≤ 14 days before SARS-CoV-2 test			–	
Always, often	36 (90.0)	80 (100.0)		
Sometimes, rarely, never	4 (10.0)	0 (0.0)		
Known exposure to a COVID-19 case or someone with signs/symptoms suggestive of COVID-19 ≤ 14 days before SARS-CoV-2 test	19 (47.5)	10 (12.5)	6.21 (2.29–16.87)	< 0.001

COVID-19, Coronavirus disease 2019; OR, odds ratio. CI: confidence interval.

^*^Univariable conditional logistic regression model for factors associated with SARS-CoV-2 infection.

Results are expressed as mean (standard deviation, SD), median (interquartile range, IQR), or frequency (percentage).

As for other potential exposures in the 2 weeks before testing, cases and controls took part in all the activities investigated to a similar extent, with the only exception being activities inside the university campus, which were attended by a greater proportion of students in the control group, although this did not reach statistical significance (35.0 vs. 17.5%, *p* = 0.067) (Table 3). Indeed, no meaningful difference was observed for visiting bars or restaurants within or outside the university campus; visiting cinemas, theaters, museums, or churches; visiting salon or aesthetic centers, shopping centers, or grocery stores; taking part in volunteer activities or courses outside the university; being or having visitors in home/social or religious gatherings; taking part in indoor sport activities; using public transportation for either short or long distances; and attending healthcare facilities.

**Table 3:** Students' activity-related exposures ≤ 14 days before testing for SARS-CoV-2.

	**Cases**	**Controls**	**Unadjusted OR** (95% CI) *	*p*-Value*
Activities inside the university campus	7 (17.5)	28 (35.0)	0.43 (0.18–1.06)	0.067
Bar/restaurants inside the university campus	8 (20.0)	24 (30.0)	0.57 (0.22–1.47)	0.243
Bar/restaurants outside the university campus	28 (70.0)	56 (70.0)	1.00 (0.40–2.48)	0.999
Cinemas, theaters, museums, churches	3 (7.5)	11 (13.8)	0.48 (0.12–1.41)	0.294
Salon/aesthetic centers, shopping centers, grocery stores, banks, post offices	23 (57.5)	55 (68.8)	0.64 (0.30–1.37)	0.248
Volunteer activities or extra-university courses	2 (5.0)	7 (8.8)	0.57 (0.12–2.75)	0.485
Visitors in home/private social or religious gatherings	30 (75.0)	54 (67.5)	1.46 (0.61–3.50)	0.398
Indoor sport activities	1 (2.5)	0 (0.0)	–	–
Use of public transportation for short distances (bus, metro, car sharing)	24 (60.0)	51 (63.8)	0.84 (0.36–1.92)	0.672
Use of public transportation for long distances (airplane, boat, interregional/international train or buses)	11 (27.5)	20 (25.0)	1.13 (0.49–2.62)	0.773
Healthcare facilities (general practitioner, hospital, other)	11 (27.5)	17 (21.3)	1.45 (0.57–3.68)	0.431

OR, odds ratio; CI, confidence interval.

^*^Univariable conditional logistic regression model for factors associated with SARS-CoV-2 infection.

Results are expressed as frequency (percentage).

In the multivariable analysis, a known exposure to a COVID-19 case or someone with sign/symptoms suggestive of COVID-19 in the 2 weeks before testing increased the likelihood of SARS-CoV-2 infection (aOR: 8.39, 95% CI: 2.38–29.54), while having a job (aOR: 0.23, 95% CI: 0.06–0.88) was negatively associated with the outcome (Table 4, Model 1). Activities in the university and having or being visitors/attending social or religious gatherings were close to statistical significance. In particular, students attending activities inside the university campus seemed less likely to become infected (aOR: 0.32, 95% CI: 0.09–1.06), whereas students attending private social or religious gatherings seemed more likely to be SARS-CoV-2 positive (aOR: 3.48, 95% CI: 0.95–12.79). Age, gender, activities in the community, eating at bar or restaurants, and use of public transportation were not predictors of SARS-CoV-2 infection. The results of the second model, which was restricted to students who did not report attending the university campus in the 2 weeks before testing, were comparable to the first analysis (Table 4, Model 2). Specifically, a known exposure to a COVID-19 case or someone with signs/symptoms suggestive of COVID-19 was the strongest predictor of SARS-CoV-2 acquisition (aOR: 57.21, 95% CI: 2.48–1,320.26), whereas having a job reduced the risk of infection (aOR: 0.05, 95% CI: 0.01–1.01). Conversely, activities in the community were almost significant (aOR: 8.13, 95% CI: 0.91–72.84). None of the other variables showed any meaningful association with the outcome.

**Table 4:** Multivariable conditional logistic regression model for SARS-CoV-2 infection among Sapienza University students (Model 1) or restricted to those students that did not report attending the university campus in the 2 weeks before testing (Model 2).

	**Model 1**		**Model 2**	
	**aOR (95% CI)**	***p*-Value**	**aOR (95% CI)**	***p*-Value**
Age (years)	0.89 (0.74–1.06)	0.198	0.74 (0.49–1.10)	0.130
Gender (female)	0.73 (0.27–1.97)	0.536	0.30 (0.05–1.84)	0.193
Having a job (yes)	0.23 (0.06–0.88)	0.033	0.05 (0.01–1.01)	0.051
Known exposure to a COVID-19 case or someone with signs/symptoms suggestive of COVID-19 (yes)	8.39 (2.38–29.54)	0.001	57.21 (2.48–1,320.26)	0.012
Activities in the community (yes)	1.21 (0.42–3.49)	0.725	8.13 (0.91–72.84)	0.061
Activities inside the university campus (yes)	0.32 (0.09–1.06)	0.062	–	–
Bar or restaurants (yes)	0.93 (0.27–3.19)	0.910	0.82 (0.13–5.19)	0.836
Use of public transportation (yes)	0.73 (0.23–2.36)	0.598	0.32 (0.05–2.07)	0.230
Visitors in home/private social or religious gatherings (yes)	3.48 (0.95–12.79)	0.060	3.36 (0.28–40.0)	0.338

aOR, adjusted Odds Ratio; CI, confidence interval.

## Discussion

In the early months of 2020, universities were left with no choice but to adapt to school closure policies and convert to emergency virtual learning (6). However, as the summer approached, governments became concerned about the loss of learning that occurred in the previous months and urged immediate action, including reopening schools (21). To safely welcome back students in September 2020, Sapienza University developed a layered approach that included a strong communication campaign on the four basic rules for infection prevention (hand washing, stay at home if showing symptoms, physical distancing, and mask use) (22) and contributed to safe learning environments, minimized campus transmission and outbreaks, and allowed the resumption of in-person activities. In this context, the voluntary testing program, in addition to its relevance to the test-trace-isolate-quarantine strategy (23), represented a key opportunity for students to reduce any anxiety around the risk of getting the infection or infecting their loved ones. Indeed, the COVID-19 pandemic has dramatically impacted the psychological wellbeing of students worldwide (24), including in Italy (25), and the offer of a SARS-CoV-2 test free of charge has been welcomed in several other universities that implemented a similar program (8, 26). In this regard, the fact that the greatest proportion of students willing to be tested registered for the program when the number of cases was still high and the vaccination campaign was in its early stages (27) likely confirms the psychological benefits of offering such a service at a critical time in the pandemic trajectory.

Although our case detection rate was generally lower than that observed at the regional level, probably because individuals had to be asymptomatic at the time of the test, the overall trends were comparable. As mentioned above, Sapienza is one of the largest universities in Europe by number of enrollments (18). It is located in the metropolitan area of Rome, the capital of the Lazio region, which with its almost three million inhabitants represents the most populous city in Italy (28). The similar trend in infection rates between schools and the surrounding communities was initially interpreted as evidence that the former made no contribution to the spread of SARS-CoV-2 (29, 30). However, it has now become clear that the reopening of schools does impact community infection rates, even though appropriate mitigation strategies reduce this effect (30). There is no doubt that university testing can be effective at limiting the spread of the virus in this setting (31, 32), especially when contact tracing has fast turnaround times, as in our study. In addition, the testing center became an educational site for students to gain clinical hours as part of their internships. Since hospitals limited access to their facilities during the pandemic to reduce the number of people exposed to the virus, such a program became a useful way for students to enhance their education in infection prevention and control and to develop adequate knowledge and skills on the provision of care during a pandemic (8).

Another advantage of the testing program was the opportunity to implement a nested case-control study. In this investigation, participants with and without SARS-CoV-2 reported generally similar rates of exposures, leading us to hypothesize that the risk of transmission may be low in places in which strict mitigation strategies are implemented. As for exposure within the community, despite bars and restaurants being widely recognized as risk factors for SARS-CoV-2 acquisition (12, 33), in our study the presence of strict public health measures, such as a limitation on the number of diners allowed and a continuation of a curfew requiring individuals to return to their residences by 10 PM, coupled with the fact that students started to eat outside as spring progressed, may have contained the spread of the virus (34–36). Similarly, previous research conducted in France during October-November 2020 (12) has documented how public transportation may not have accelerated transmission during stay-at-home orders: only a few people were traveling long distances and all were subjected to seat arrangement strategies and rigorous mask wearing; a higher number of individuals made short journeys, but with limited interaction between passengers, a factor that reduces the opportunity for viral infections (37). However, as expected (38, 39), in our study participants with confirmed SARS-CoV-2 infection were much more likely than individuals without the virus to have reported close contact with a COVID-19 case or someone with influenza-like illness. It may be not a coincidence that most close contacts usually occur in the household setting, where it is more difficult to implement preventive measures (38). Interestingly, while in-office working has commonly been associated with SARS-CoV-2 infection in the general population (11, 12), our students with a part- or full-time job had a lower likelihood of contracting the virus. A possible explanation is that working students usually come from low-income settings and, since they cannot afford to become infected and lose further work days in addition to those already lost in lockdown periods (40), they may have been particularly careful in adopting preventive behaviors in general.

Among the other potential exposures that occurred in the 2 weeks before COVID-19 testing, only two factors came close to statistical significance. First, taking part in private gatherings, at home or elsewhere, almost increased the likelihood of infection, suggesting that settings in which preventive measures can be partially or fully ignored may contribute to the spread of the virus (41). By contrast, students regularly attending lectures or internships inside the university campus seemed less likely to become infected. This potentially protective effect may be explained by the fact that these students were more exposed to the Sapienza communication campaign on the four basic rules that improve collective safety (22) and, therefore, they were also less likely to adopt risky behavior outside the university. This consideration may also explain why in the analysis of non-attending students only, activities in the community almost significantly increased the risk of infection, suggesting that individual behavior may play a role even in those settings where rigorous measures are in place. Nevertheless, since the testing program was voluntary, we cannot exclude the possibility that those students attending the university campus were more likely to get tested for screening purposes (i.e., with low or no likelihood of COVID-19) compared to non-attending students, even though this bias could be counteracted if those individuals that were tested were overcautious, or if exposed individuals avoided testing because they did not want to be subject to isolation (42). However, in a secondary analysis where attending students were excluded from the model, such that all study participants had the same exposure conditions, our conclusions did not change meaningfully.

This study has several strengths and limitations. To the best of our knowledge, this is one of the few studies that investigates behavioral risk factors for SARS-CoV-2 infection in a specific population that is often asymptomatic and is highly sociable. Moreover, by adopting a test-negative study design, we were able to rule out asymptomatic infections in controls, which would have distorted the association of interest. In addition, we enrolled incident cases that were later matched by calendar time to controls, meaning that both groups were exposed to the same mitigation measures. Conversely, there are potential information biases in this study, such as social desirability and recall bias. Since interviews were conducted after the test result, it may have influenced the students' answers. Secondly, even though we achieved a good response rate, the limited sample size may have led to reduced statistical power. In addition, since we were only able to adjust our models for a few variables, residual confounding cannot be excluded. Lastly, the opt-in procedure for the testing program may mean our students were unrepresentative of the general Sapienza University population, especially given that most international students were still living in their own country at the time of the study. Therefore, these findings should be interpreted in the context of the restrictions and public health measures that were implemented in the Lazio region in the spring of 2021, which also included the vaccination campaign that was in its initial stages for young adults. For these reasons and given also the emergence of more transmissible variants and the relaxation of mitigation measures, additional research is needed to better investigate the behavioral risk factors for SARS-CoV-2 acquisition in this sub-group.

The findings of this study, which are consistent with our knowledge of SARS-CoV-2 transmission, endorse infection prevention and control measures specific to this virus (12). We documented how a testing program was effectively and efficiently carried out in the university setting and contributed to creating a safe learning and working environment. In addition, we showed that places in which rigorous adherence to SARS-CoV-2 infection prevention and control measures was implemented, such as the university setting, did not increase the likelihood of infection. Since young adults frequently engage in social interaction and are highly mobile, these findings could be used to guide public health measures and develop tailored strategies in those contexts that are struggling with high infection rates.

## Data availability statement

The raw data supporting the conclusions of this article will be made available by the authors, without undue reservation.

## Ethics statement

The studies involving human participants were reviewed and approved by Policlinico Umberto I Teaching Hospital/Sapienza University of Rome (prot. 188/2021). The patients/participants provided their written informed consent to participate in this study.

## Collaborating group

Federica Maria Di Lella^1^, Sofia Di Virgilio^2^, Pierluigi Donia^3^, Emiliano Rapiti^2^, Donatella Maria Rodio^1^, Geltrude Taddeo^3^

^1^ Microbiology and Virology Unit, Policlinico Umberto I Teaching Hospital, Sapienza University of Rome, Rome, Italy

^2^ Special Office for Prevention, Protection and High Vigilance, Sapienza University of Rome, Rome, Italy

^3^ Department of Public Health and Infectious Diseases, Sapienza University of Rome, Rome, Italy

## Author contributions

VB, ERe, GM, PV, and AP contributed to the conception and design of the study. LS, ERe, CC, AR, AM, FD, SV, PD, DMR, ERa, and GT performed data collection. VB, LS, and ERe conducted the analyses. LS and GM contributed to data curation. VB wrote the first draft of the manuscript. LS and ERe wrote sections of the manuscript. CM, CD, LC, GA, OT, AA, FD'A, PV, and AP critically revised the manuscript. LC, GA, OT, AA, FD'A, PV, and AP contributed to supervision and funding acquisition. All authors contributed to manuscript revision and read and approved the submitted version.

## Conflict of interest

The authors declare that the research was conducted in the absence of any commercial or financial relationships that could be construed as a potential conflict of interest.

## Publisher's note

All claims expressed in this article are solely those of the authors and do not necessarily represent those of their affiliated organizations, or those of the publisher, the editors and the reviewers. Any product that may be evaluated in this article, or claim that may be made by its manufacturer, is not guaranteed or endorsed by the publisher.
